# Health and adverse events associated with extended oral bisphosphonates among postmenopausal women: a systematic review

**DOI:** 10.1210/clinem/dgag057

**Published:** 2026-02-10

**Authors:** Prawira Oka, Aminath Shiwaza Moosa, Eileen Yi Ling Koh, Chirk Jenn Ng

**Affiliations:** Department of Research, SingHealth Polyclinics, Singapore 150167, Singapore; SingHealth-Duke NUS Family Medicine Academic Clinical Program, Singapore 169857, Singapore; Department of Research, SingHealth Polyclinics, Singapore 150167, Singapore; SingHealth-Duke NUS Family Medicine Academic Clinical Program, Singapore 169857, Singapore; Department of Research, SingHealth Polyclinics, Singapore 150167, Singapore; Department of Research, SingHealth Polyclinics, Singapore 150167, Singapore; SingHealth-Duke NUS Family Medicine Academic Clinical Program, Singapore 169857, Singapore

**Keywords:** bisphosphonate, fracture, menopause, osteoporosis, women

## Abstract

**Background:**

International guidelines recommend the use of oral bisphosphonates (oBP) for up to 5 years in the treatment of postmenopausal osteoporosis. However, benefits and risks of extending oBP therapy beyond this duration remain uncertain.

**Aim:**

To determine the health outcomes (bone mineral density [BMD], fracture risk) and adverse effects (atypical fracture, osteonecrosis of the jaw [ONJ]) of extended oBP therapy beyond 5 years among postmenopausal women.

**Methodology:**

A systematic search of Medline©, EMBASE©, and CINAHL© was conducted from inception to 15 March 2024. Interventional and observational studies published in English were included. Two authors independently performed screening, quality appraisal using the NIH Quality Appraisal tool, and data extraction. The review followed PRISMA guidelines and was registered on PROSPERO (CRD42024475332). High heterogeneity precluded meta-analysis.

**Results:**

Eleven studies (4 controlled trials, 7 observational) were included: 3 rated as good quality, 7 as fair, and 1 as poor. Extended oBP therapy beyond 5 years increased BMD at the hip (3/3 studies), femoral neck (3/3 studies) and lumbar spine (5/5 studies). Prolonged oBP use reduced clinical vertebral fracture risk (1/6 studies) but increased the risk of atypical fractures (3/3 studies) and incomplete atypical femoral fractures (1/1 study). None of the included studies assessed or reported ONJ.

**Conclusion:**

Substantial heterogeneity and limited high-quality evidence preclude definitive conclusions regarding extended oBP use in postmenopausal women. While extended treatment may improve bone density and reduce vertebral fractures, it could also increase atypical fractures. Future high-quality studies are required to inform decisions regarding optimal treatment duration.

Osteoporosis is a chronic metabolic bone disease characterized by reduced bone density and increased fracture risk (1). Its prevalence increases with age, impacting one-third of adults aged 50-60 years and more than half of those over 80 years (2). The condition disproportionately impacts women, affecting 1 in 4, nearly twice the prevalence in men (3). Vertebral, wrist, and hip fractures are among the significant complications associated with this disease, with hip fractures associated with a 1-year mortality rate of 30% (4). Aside from mortality, osteoporotic fractures are also associated with increased morbidity, reduced quality of life, and significant healthcare costs (5, 6).

Effective osteoporosis treatment with antiresorptive agents has been shown to substantially reduce the risk of vertebral fractures by 45% (7), morbidity (8), and mortality by 11% (9). Apart from antiresorptive agents, sequential therapy with osteoanabolic agents such as romosozumab has demonstrated further reductions in vertebral fracture risk (10). The choice between initiating antiresorptive and osteoanabolic agents depends on a patient's fracture risk. According to the International Osteoporosis Foundation and multiple national guidelines, oral bisphosphonates (oBP) are recommended as first-line therapy for postmenopausal women with high fracture risk, while osteoanabolic agents are indicated for those at very high risk of fractures, defined as those with very low bone mineral density (BMD; *T*-score ≤ −3.0) and multiple vertebral or fragility fractures (11–15). These agents reduce bone turnover through reducing resorption, resulting in increased BMD and a consequent reduction in osteoporotic fractures during the initial 5 years of therapy (16).

However, the benefits of bisphosphonate therapy beyond 5 years remain uncertain (17). While extended therapy has shown beneficial effects in reducing osteoporotic fracture risk (18, 19), it has also been associated with rare adverse events such as atypical femoral fractures (AFF) and osteonecrosis of the jaw (ONJ) (20–22). Studies have shown that long-term bisphosphonate use may result in the degradation of the fracture-resistance toughening mechanisms, thereby increasing the risk of AFF (23). In contrast, the pathophysiology of ONJ remains incompletely understood; proposed mechanisms include suppressed bone remodeling, antiangiogenic effects, and oral mucosa toxicity, which may increase susceptibility to oral mucosa injury and infection, culminating in ONJ (24). Current clinical guidelines provide limited clarity on the risk-benefit profile of extending oBP therapy beyond 5 years, necessitating physicians make these decisions on an individual basis (25, 26). These uncertainties culminate in potential therapeutic dilemmas, especially in the primary care setting, where the majority of patients with postmenopausal osteoporosis are treated with oBP (27).

Recent publications have attempted to address the clinical dilemma associated with extended bisphosphonate therapy. A systematic review by Fink et al found that long-term alendronate reduced fracture risk in women with osteoporosis but might increase the risk of atypical fractures (28). However, the systematic review selected a 3-year cut-off when defining long-term treatment and pooled data from male and female participants, thereby limiting its applicability to postmenopausal women (28). Similarly, while Gedmintas et al's review found increased risk of atypical fractures with more than 5 years of bisphosphonate therapy, the pooled data included both genders and intravenous BP, further limiting its relevance to deciding whether oBP should be extended among postmenopausal women (29). Therefore, this review focused specifically on oBP, as these agents represent the most commonly prescribed first-line treatment for postmenopausal osteoporosis in primary care, where the majority of cases are managed. Moreover, intrinsic differences in bioavailability, pharmacokinetics, and adherence patterns between the 2 formulations could introduce heterogeneity and reduce generalisability of the findings.

To address this uncertainty, this systematic review aimed to determine the health outcomes (BMD, osteoporotic fractures) and adverse events (atypical fractures, osteonecrosis of jaw) associated with extended oBP use beyond 5 years in postmenopausal women.

## Methods

A protocol detailing the search methods employed was registered on PROSPERO (CRD42024475332) (https://www.crd.york.ac.uk/PROSPERO/view/CRD42024475332). This systematic review was conducted and reported in accordance with the Preferred Reporting Items for Systematic Reviews and Meta-Analyses (PRISMA) reporting guidelines (30).

### Data sources

A comprehensive search of records published from inception to 15 March 2024 was conducted using the electronic databases: PubMed/Medline©, EMBASE©, and CINAHL©. These databases were selected due to their comprehensive and complementary coverage of records relevant to the review question. Given the large number of eligible records generated by the preliminary search, additional databases were not included. To enhance completeness, backward and forward reference searching was performed on all included reports.

### Search strategy

The authors developed the search strategy in consultation with an experienced librarian. It combined MeSH and key terms relating to the following 3 key concepts: (1) postmenopausal osteoporosis, (2) bisphosphonates, and (3) health outcomes or adverse effects.

The key concepts used the following controlled vocabulary (MeSH, Emtree, CINAHL subject headings) and key terms: “Bone Diseases, Metabolic”[MeSH], “postmenopause,” “diphosphonates”[MeSH], “fractures, bone”[MeSH], and “osteonecrosis”[MeSH]. Boolean operators were utilized to combine these key concepts across databases.

### Postmenopausal osteoporosis

For the purposes of this review, postmenopausal osteoporosis was defined as progressive bone loss following the onset of natural or surgical menopause (31). The definition was deliberately selected to reflect clinical practice, where osteoporosis treatment is also indicated for women with osteopenia-range BMD measurements and elevated fracture risk. Including these individuals was therefore essential to achieve the review's objective of evaluating the potential benefits and harms associated with extended oBP use.

### Data collection and study selection

All records were exported to Covidence®, a web-based collaboration platform designed to streamline systematic reviews (32). Duplicates were removed through Covidence® and manual review before screening. The titles and abstracts of the exported records were independently screened by 2 authors per the inclusion and exclusion criteria. The full texts of the screened articles were retrieved and assessed for eligibility. Any discrepancies were resolved by a third author throughout the screening process.

### Inclusion criteria

Observational and interventional studies published in English involving postmenopausal women with osteoporosis receiving oBP beyond 5 years were included. Observational studies were included to supplement the limited availability of interventional studies addressing the review question, especially with regard to the adverse effects of extended oBP therapy. To maintain consistency in the study designs of the included studies across the different outcomes, acceptable studies included randomized and nonrandomized trials, cohort, and case-control studies.

### Exclusion criteria

Qualitative studies, editorials, reviews, letters, commentaries, cross-sectional, and case studies were excluded. Studies involving men, and oBP prescribed for neoplasm-related indications or less than 5 years were also excluded.

### Data extraction

Two authors independently conducted the extraction, with discrepancies resolved by a third author. The findings of interest were study setting, design, participants, age, ethnicity, oBP type, dose, and duration, and reported outcomes (health outcomes/adverse effects). In the event of missing or unclear information, the author of the publication was contacted for clarification.

### Outcomes

Health outcomes were defined as osteoporotic fractures and the BMD rate of change.

Adverse events were defined as ONJ and atypical femoral shaft fractures.

### Risk of bias assessment

Included studies were independently appraised by 2 authors, with discrepancies resolved by a third author. The selected articles were assessed using the relevant National Institute of Health (NIH) Quality Assessment Tools. These tools were designed to assess a study's internal validity, with 6 separate tools to administer based on study design (33). The full set of questions included for each tool is available on the NIH website (https://www.nhlbi.nih.gov/health-topics/study-quality-assessment-tools).

### Data synthesis

The full texts of all included studies were analyzed by 2 authors, with the key findings narratively synthesized. The study characteristics of interest included: study setting, population, study design, oBP prescribed, and reported outcomes. Studies reporting more than one outcome of interest (eg, BMD change and osteoporotic fractures) were presented under each relevant table.

The high clinical and methodological heterogeneity among the included studies precluded meta-analysis. Clinical heterogeneity stemmed from differences in oBP type, dose, and duration, while variations in study design and statistical analyses contributed to methodological heterogeneity.

## Results

### Study selection

**Figure 1:**
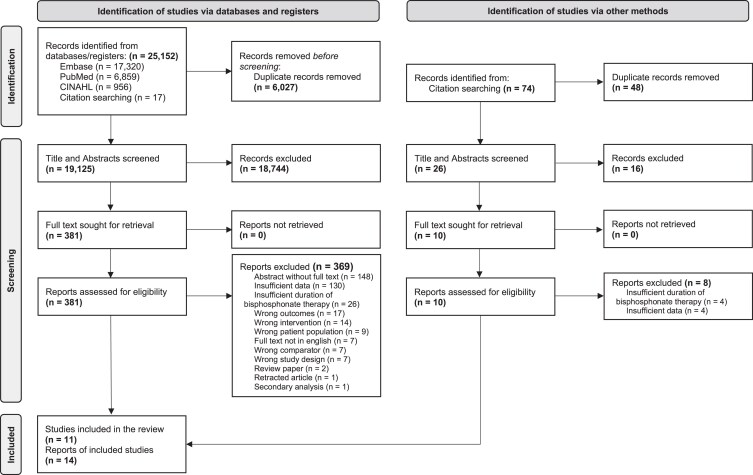
PRISMA flow diagram of study selection.

A total of 25 152 records were identified through the search strategies employed on the Medline©, EMBASE©, and CINAHL© electronic databases. After excluding duplicates, 19 125 records remained. Following title and abstract screening, 381 full texts were reviewed. A total of 11 studies and 14 reports were included in the review, of which data were extracted from 11 reports. Forward and backward citation searches conducted on the 11 included studies yielded 2 new publications (Fig. 1) (34, 35). Data from 3 reports were not extracted as they were a pre-extension of an included report (34, 36), or presented post hoc analysis of an already included study (35).

### Study characteristics

Table 1 summarizes the included studies, detailing the study design, setting, population, comparator group, outcome definition, and oBP type and dose. Of the 11 studies, there were 3 RCTs (37, 38, 44), 1 nonrandomized controlled trial (43), 4 retrospective cohort studies (18, 40, 41, 46), 1 prospective cohort (39), and 2 case-control studies (42, 45). Five studies were conducted in the United States (18, 37, 38, 40, 44), and one study each from Canada (45), Japan (41), South Korea (42), Taiwan (46), and Turkey (39); the setting was unclear in one study (43). All studies involved postmenopausal women, with 2 including only women with existing vertebral fractures (43, 44).

**Table 1:** Overview of included studies

Study ID	Study design	Country	Participants, age, ethnicity	Osteoporosis diagnosis	Previous osteoporotic fractures	Intervention/case	Control/comparator(s)	Outcome(s)
Black 2006 (37)	RCT	USA	1099 Pm Mean age: 73 Ethnicity: White (97.3%)	Low femoral neck BMD (<0.68 g/cm^2^)	ALN × 5 years + PBO × 5 years: 260 (59.5%) ALN 5 mg × 10 years: 196 (59.6%) ALN 10 mg × 10 years: 204 (61.3%)	ALN (5 & 10 mg)×10 years	ALN × 5 years + PBO × 5 years	Mean BMD % difference Osteoporotic fractures
Bone 2004 (38)	RCT	USA/others	994 Pm Mean age: 63 Ethnicity: unspecified	Lumbar spine BMD	ALN 10 mg × 10 years: 17.5% ALN 5 mg × 10 years: 30.8% ALN 20 mg × 2 years + ALN 5 mg × 3 years + PBO × 5 years: 27.2%	ALN 10 mg × 10 years	ALN 5 mg × 10 years ALN 20 mg × 2 years + ALN 5 mg × 3 years + PBO × 5 years	Mean BMD % difference Morphometric vertebral fracture
Canbek 2019 (39)	Cohort	Turkey	132 Pm with osteoporosis Mean age: 72.8 Ethnicity: unspecified	Part of inclusion criteria but not clearly defined	None, fracture history was part of exclusion criteria	oBP ≥ 10 years	oBP ≤ 4 years - oBP × 5-9 years	iAFF
Drieling 2017 (40)	Retrospective cohort	USA	5120 Pm [4369 without cancer] Mean age: 80 Ethnicity: 94.3% White	Predicted 5-year risk of hip fracture ≥1.5% (comparable to high FRAX risk)	No baseline data	oBP × 2 years	oBP × 3-5 years oBP × 6-9 years - oBP × 10-13 years	Osteoporotic fractures
Iwamoto 2010 (41)	Retrospective cohort	Japan	47 Pm Mean age: 65.7 Ethnicity: Japanese (100%)	Japanese osteoporosis diagnostic criteria	Vertebral fracture: 23 (48.9%)	ALN 5 mg daily or 35 mg weekly × 7 years	None	% BMD change from baseline
Izano 2020 (18)	Retrospective cohort	USA	29 685 women aged 45-80 years old Median age: 71 Ethnicity: White (60%), Asian (20%), Hispanic (13%), African American (4%)	Not clearly defined	Hip fracture: 666 (2%) other major osteoporotic fracture: 3384 (11%) any clinical fracture: 8224 (28%)	oBP × 10 years	oBP × 5 years oBP × 7 years	Hip fracture
Koh 2017 (42)	Case-control	South Korea	172 Pm Mean age: 68.1 Ethnicity: unspecified	Not clearly defined	Case: 8 (18.6%) Control: 15 (11.6%)	AFF	No AFF	AFF
Mellström 2004 (43)	CT	Unclear	164 Pm Mean age: 68.8 Ethnicity: unspecified	Pre-existing vertebral fracture	≥2 vertebral fractures	RIS × 7 years	PBO × 5 years + RIS × 2 years	Mean BMD % difference Osteoporotic fractures
Miller 1997 (44)	RCT	USA	263 Pm Mean age: 70.4 Ethnicity: Caucasian/Asian, data not provided	Pre-existing vertebral fracture	1-4 vertebral fractures	ETI × 7 years	ETI × 5 years ETI × 4 years ETI × 2 years	Mean % BMD change from baseline Osteoporotic fractures
Park-Wyllie 2011 (45)	Case-control	Canada	4296 Pm on oBP Median age: 83 Ethnicity: unspecified	Not clearly defined	Case: 500 (69.8%) Control: 861 (24.1%)	ST/femoral shaft fracture	No ST/femoral shaft fracture	ST/femoral shaft fracture
Wang 2016 (46)	Retrospective cohort	Taiwan	1342 women aged ≥50 years old Mean age: 71 Ethnicity: unspecified	ICD-9-CM coded diagnosis	No baseline data	oBP ≥ 5 years	oBP < 5 years	Osteoporotic fractures ST/diaphyseal fractures

Abbreviations: AFF, atypical femoral fracture; ALN, alendronate; ETI, etidronate; iAFF, incomplete AFF; ICD-9-CM, International Classification of Diseases, 9th Revision, Clinical Modification; oBP, oral bisphosphonates; PBO, placebo; PM, postmenopausal women; RIS, risedronate; ST, subtrochanteric.

Among the 4 controlled trials, only Black et al directly compared the effect of 5 years of oBP against continuation at 10 years (37). The remaining 3 controlled trials only performed within-group analysis (38, 43, 44). The 4 studies reporting AFF employed differing definitions. One study defined AFF in accordance with the American Society for Bone and Mineral Research criteria (42), 2 studies included subtrochanteric and diaphyseal fractures via ICD-9 (46) or ICD-10 codes (45), and one study reported incomplete AFF based on increased isotope uptake on bone scintigraphy (39). The studies also varied in the type of oBP. Three studies utilized alendronate (37, 38, 41), 1 used risedronate (43), 1 used etidronate (44), with the remaining 6 studies using mixed oBP (18, 39, 40, 42, 45, 46). Additionally, 3 studies involved a subtherapeutic alendronate dose (5 mg) for osteoporosis in one comparison group (37, 38, 41)

### Quality of included studies

Overall, 3 studies were of good quality (18, 37, 39), 1 was poor (43), and the remaining 7 were of fair overall quality (38, 40–42, 44–46). Table 2 summarizes the 4 included controlled trials. Only Black et al had a good overall rating (37), with 2 studies rated as fair (38, 44), and another poor (43). Mellström et al was rated poor due to a lack of randomization, drop-out reporting, and per protocol analysis. For most studies, it was challenging to ascertain adequacy of randomization, allocation concealment, outcome assessor blinding, and sample size calculation because the study protocol were unavailable or lacked the required information despite attempting to search clinicaltrials.gov and contacting the authors.

**Table 2:** Controlled intervention studies graded by NIH quality assessment tool

No.	Publication	Q1	Q2	Q3	Q4	Q5	Q6	Q7	Q8	Q9	Q10	Q11	Q12	Q13	Q14	Quality rating
1	Black 2006 (37)	Y	CD	CD	Y	Y	Y	Y	Y	N	Y	Y	Y	Y	Y	GOOD
2	Bone 2004 (38)	Y	CD	CD	Y	NR	Y	N	Y	NR	N	Y	NR	Y	Y	FAIR
3	Mellström 2004 (43)	N	NA	NA	Y	CD	Y	N	Y	Y	Y	Y	NR	Y	N	POOR
4	Miller 1997 (44)	Y	CD	NR	Y	CD	Y	Y	Y	Y	Y	Y	NR	Y	N	FAIR

Each study was assessed for quality with the NIH Quality Assessment Tool by 2 independent authors. Differences in grading were resolved by an arbitrator. The final grading of each article on each question is shown here.

Abbreviations: CD, cannot determine; N, no; NR, not reported; NA, not applicable; Y, yes.

Q1. Was the study described as randomized, a randomized trial, a randomized clinical trial, or an RCT?

Q2. Was the method of randomization adequate (ie, use of randomly generated assignment)?

Q3. Was the treatment allocation concealed (so that assignments could not be predicted)?

Q4. Were study participants and providers blinded to treatment group assignment?

Q5. Were the people assessing the outcomes blinded to the participants’ group assignments?

Q6. Were the groups similar at baseline on important characteristics that could affect outcomes (eg, demographics, risk factors, comorbid conditions)?

Q7. Was the overall drop-out rate from the study at endpoint 20% or lower of the number allocated to treatment?

Q8. Was the differential drop-out rate (between treatment groups) at endpoint 15% points or lower?

Q9. Was there high adherence to the intervention protocols for each treatment group?

Q10. Were other interventions avoided or similar in the groups (eg, similar background treatments)?

Q11. Were outcomes assessed using valid and reliable measures, implemented consistently across all study participants?

Q12. Did the authors report that the sample size was sufficiently large to be able to detect a difference in the main outcome between groups with at least 80% power?

Q13. Were outcomes reported or subgroups analyzed prespecified (ie, identified before analyses were conducted)?

Q14. Were all randomized participants analyzed in the group to which they were originally assigned, ie, did they use an intention-to-treat analysis?

Table 3 presents the 5 cohort studies included in this review.

**Table 3:** Cohort studies graded by NIH quality assessment tool

No.	Publication	Q1	Q2	Q3	Q4	Q5	Q6	Q7	Q8	Q9	Q10	Q11	Q12	Q13	Q14	Quality rating
1	Canbek 2019 (39)	Y	Y	Y	Y	Y	Y	Y	Y	Y	N	Y	NR	Y	Y	GOOD
2	Drieling 2017 (40)	Y	Y	Y	Y	N	Y	Y	Y	Y	N	Y	N	NA	Y	FAIR
3	Iwamoto 2010 (41)	Y	Y	Y	Y	N	Y	Y	N	Y	N	Y	NR	NA	N	FAIR
4	Izano 2020 (18)	Y	Y	Y	Y	N	Y	Y	Y	Y	Y	Y	N	NA	Y	GOOD
5	Wang 2016 (46)	Y	Y	Y	Y	N	Y	Y	Y	Y	N	Y	N	NA	Y	FAIR

Each study was assessed for quality with the NIH Quality Assessment Tool by 2 independent authors. Differences in grading were resolved by an arbitrator. The final grading of each article on each question is shown here.

Abbreviations: CD, cannot determine; N, no; NR, not reported; NA, not applicable; Y, yes.

Q1. Was the research question or objective in this paper clearly stated?

Q2. Was the study population clearly specified and defined?

Q3. Was the participation rate of eligible persons at least 50%?

Q4. Were all the subjects selected or recruited from the same or similar populations (including the same time period)? Were inclusion and exclusion criteria for being in the study prespecified and applied uniformly to all participants?

Q5. Was a sample size justification, power description, or variance and effect estimates provided?

Q6. For the analyses in this paper, were the exposure(s) of interest measured prior to the outcome(s) being measured?

Q7. Was the timeframe sufficient so that one could reasonably expect to see an association between exposure and outcome if it existed?

Q8. For exposures that can vary in amount or level, did the study examine different levels of the exposure as related to the outcome (eg, categories of exposure, or exposure measured as continuous variable)?

Q9. Were the exposure measures (independent variables) clearly defined, valid, reliable, and implemented consistently across all study participants?

Q10. Was the exposure(s) assessed more than once over time?

Q11. Were the outcome measures (dependent variables) clearly defined, valid, reliable, and implemented consistently across all study participants?

Q12. Were the outcome assessors blinded to the exposure status of participants?

Q13. Was loss to follow-up after baseline 20% or less?

Q14. Were key potential confounding variables measured and adjusted statistically for their impact on the relationship between exposure(s) and outcome(s)?

Two studies had a good overall rating (18, 39), with the remaining 3 deemed to be of fair quality (40, 41, 46).

Table 4 summarizes the 2 included case-control studies; both were deemed to be of fair quality (42, 45).

**Table 4:** Case-control studies graded by NIH quality assessment tool

No.	Publication	Q1	Q2	Q3	Q4	Q5	Q6	Q7	Q8	Q9	Q10	Q11	Q12	Quality rating
1	Koh 2017 (42)	Y	Y	N	Y	Y	Y	N	Y	Y	Y	N	Y	FAIR
2	Park-Wyllie 2011 (45)	Y	Y	N	Y	Y	Y	N	Y	Y	Y	N	Y	FAIR

Each study was assessed for quality with the NIH Quality Assessment Tool by 2 independent authors. Differences in grading were resolved by an arbitrator. The final grading of each article on each question is shown here.

Abbreviations: CD, cannot determine; N, no; NR, not reported; NA, not applicable; Y, yes.

Q1. Was the research question or objective in this paper clearly stated and appropriate?

Q2. Was the study population clearly specified and defined?

Q3. Did the authors include a sample size justification?

Q4. Were controls selected or recruited from the same or similar population that gave rise to the cases (including the same timeframe)?

Q5. Were the definitions, inclusion and exclusion criteria, algorithms or processes used to identify or select cases and controls valid, reliable, and implemented consistently across all study participants?

Q6. Were the cases clearly defined and differentiated from controls?

Q7. If less than 100% of eligible cases and/or controls were selected for the study, were the cases and/or controls randomly selected from those eligible?

Q8. Was there use of concurrent controls?

Q9. Were the investigators able to confirm that the exposure/risk occurred prior to the development of the condition or event that defined a participant as a case?

Q10. Were the measures of exposure/risk clearly defined, valid, reliable, and implemented consistently (including the same time period) across all study participants?

Q11. Were the assessors of exposure/risk blinded to the case or control status of participants?

Q12. Were key potential confounding variables measured and adjusted statistically in the analyses? If matching was used, did the investigators account for matching during study analysis?

### Health outcomes

#### BMD change

The 5 studies reporting the impact of extended oBP use on BMD are summarized in Table 5 (37, 38, 41, 43, 44).

**Table 5:** Effect of extended oral bisphosphonate therapy (>5 years) on BMD change

Study, country, study design	Outcome
**Black 2006 (** 37)**, USA, RCT** Exposure vs comparator: ALN continuation (10 years ALN) vs ALN discontinuation (5 years ALN + 5 years placebo)	Mean difference, (95% CI), pooled ALN 5/10 mg
	Total hip (*n* = 1071): 2.36 (1.81-2.90)*^a^*
	Femoral neck (n = 1071): 1.94 (1.20-2.68)*^a^*
	Trochanter (n = 1071): 3.17 (2.49-3.84)*^a^*
	Lumbar spine (n = 1023): 3.74 (3.03-4.45)*^a^*
	Total body (n = 1026): 1.28 (0.70-1.86)*^a^*
	Forearm (n = 396): 2.01 (1.35-2.68)*^a^*
**Bone 2004 (** 38)**, USA/others, RCT** Exposure vs comparator: ALN 10 mg × 10 years vs ALN 5 mg × 10 years vs ALN 20 mg × 2 years + ALN 5 mg × 3 years + PBO × 5 years	Mean % difference, (95% CI), between year 6 and 10
	Lumbar spine (n = 72-81/group) Discontinuation: 0.3 (−0.8 to 1.5) ALN 5 mg: 2.5 (1.3-3.6)*^a^* ALN 10 mg: 3.7 (2.6-4.8)*^a^*
	Femoral neck (n = 71-76/group) Discontinuation: −2.2 (−3.9 to −0.5)*^b^* ALN 5 mg: 1.0 (−0.8 to 2.7) ALN 10 mg: 0.9 (−0.8 to 2.6)
	Trochanter (n = 71-76/group) Discontinuation: −1.0 (−2.7 to 7.4) ALN 5 mg: 0.0 (−1.7 to 1.7) ALN 10 mg: 1.0 (−0.7 to 2.6)
	Total hip (n = 46-50/group) Discontinuation: −1.8 (−3.5 to −0.1)*^b^* ALN 5 mg: 0.7 (−0.9 to 2.3) ALN 10 mg: 0.8 (−0.9 to 2.4)
	Total body (n = 58-64/group) Discontinuation: −0.6 (−1.7 to 0.4) ALN5mg: −0.7 (−1.8 to 0.3) ALN10mg: 0.4 (−0.6 to 1.4)
	Distal 1/3 forearm (n = 44-49/group) Discontinuation: −2.3 (−3.8 to −0.8)*^c^* ALN5mg: −0.4 (−1.8 to 1.0) ALN10mg: −0.1 (−1.6 to 1.3)
**Mellström 2004 (** 43)**, unclear, CT** Exposure vs comparator: RIS × 7 years vs PBO × 5 years followed by RIS × 2 years	Mean % difference, year 5-7
	Lumbar spine (n = 48): 1.8
**Miller 1997 (** 44)**, USA, RCT** Exposure vs comparator: ETI × 7 years vs ETI × 5 years vs ETI × 4 years vs ETI × 2 years	Mean % change from baseline, in ETI × 7year group
	Spine At 6 years (n = 44): 0.5±0.48 At 7 years (n = 40): 1.8±0.71*^b^*
	Femoral neck At 6 years (n = 44): −0.5±0.65 At 7 years (n = 40): 0.5±0.82
	Trochanter At 6 years (n = 44): −0.3±0.61 At 7 years (n = 40): 0.4±0.76
	Distal radius At 6 years (n = 34): 0.0±1.02 At 7 years (n = 29): −1.1±1.28
**Iwamoto 2010 (** 41)**, Japan, Retrospective cohort** Exposure: ALN 5 mg daily or 35 mg weekly × 7 years	%change in BMD from baseline
	Lumbar spine At 5 years (n = 47): 9.4% At 7 years (n = 47): 12.8%

Abbreviations: ALN, alendronate; ETI, etidronate; PBO, placebo; RIS, risedronate.

^*a*^ *P*-value < .001

^*b*^ *P*-value < .05.

^*c*^ *P*-value < .01.

Among the 5 studies reporting BMD, only Black et al, the sole study with a direct placebo comparison, reported significant BMD improvements with extended alendronate therapy (10 years vs 5 years) (36). Improvements were observed at the hip (mean difference [MD]: 2.36, 95%CI: 1.81-2.90), femoral neck (MD: 1.94, 95%CI: 1.20-2.68), trochanter (MD: 3.17, 95%CI: 2.49-3.84), lumbar spine (MD: 3.74, 95%CI: 3.03-4.45), total body (MD: 1.28, 95%CI: 0.70-1.86), and forearm (MD: 2.01, 95%CI: 1.35-2.68) (37). The improvement was observed despite the pooling of data from individuals receiving subtherapeutic (5 mg) alendronate doses.

For studies reporting within group analysis, Bone et al, found alendronate 10 mg for 10 years yielded increased lumbar spine BMD between year 6 and year 10 (mean % difference: 3.7, 95%CI: 2.6-4.8); while 5 years of placebo following 5 years of alendronate resulted in significant BMD reductions in femoral neck (mean % difference: −2.2, 95%CI: −3.9 to −0.5), total hip (mean % difference: −1.8, 95%CI: −3.5 to −0.1), and distal forearm (mean % difference: −2.3, 95%CI: −3.8 to −0.8) (38). Similarly, Miller et al found that 7 years of etidronate increased spine BMD when compared to baseline (mean % change: 1.8, standard deviation: 0.71) (44). The remaining studies did not report any significant BMD changes (41, 43).

### Osteoporotic fractures

The 7 studies reporting the effect of extended oBP on osteoporotic fractures are presented in Table 6 (18, 37, 38, 40, 43, 44, 46).

**Table 6:** Effect of extended oral bisphosphonate therapy (>5 years) on osteoporotic fractures

Study, country, study design	Outcome
**Black 2006 (** 37)**, USA, RCT** Exposure vs comparator: ALN continuation (10 years ALN) vs ALN discontinuation (5 years ALN + 5 years placebo)	aRR (95% CI), pooled ALN5/10mg
	Clinical vertebral fracture: 0.45 (0.24-0.85)*^a^*
	Morphometric vertebral fracture: 0.86 (0.60-1.22)
	Any clinical fracture: 0.93 (0.71-1.21)
	Nonspine fracture: 1.00 (0.76-1.32)
	Hip fracture: 1.02 (0.51-2.10)
	Forearm fracture: 1.09 (0.62-1.96)
**Bone 2004 (** 38)**, USA/others** Exposure vs comparator: ALN10 mg × 10 years vs ALN5 mg × 10 years vs ALN20 mg × 2 years + ALN5 mg × 3 years + PBO × 5 years	%
	Morphometric vertebral fracture (year 6-10) Discontinuation: 6.6 ALN5mg: 13.9 ALN10mg: 5.0
**Mellström 2004 (** 43)**, unclear, CT** Exposure vs comparator: RIS × 7 years vs PBO × 5 years followed by RIS × 2 years	Incidence in RIS × 7 years group
	Vertebral fracture Years 4-5: 5 Years 6-7: 5
**Miller 1997 (** 44)**, USA, RCT** Exposure vs comparator: ETI × 7 years vs ETI × 5 years vs ETI × 4 years vs ETI × 2 years	n (%), between year 5 and 7
	Vertebral fracture ETI × 7 years: 1 (2.4)
**Drieling 2017 (** 40)**, USA, Retrospective cohort** Exposure: oBP × 2 years vs oBP × 3-5 years vs oBP × 6-9 years vs oBP × 10-13 years	Incidence per 1000 person years, adjusted HR (95%CI)
	Hip fracture oBP × 2 years: 6.2, 1 oBP × 3-5 years: 7.4, 1.26 (0.54-2.92) oBP × 6-9 years: 7.7, 1.60 (0.66-2.92) oBP × 10-13 years: 12.3, 2.06 (0.93-4.58)
	Wrist/forearm fracture oBP × 2 years: 13.5, 1 oBP × 3-5 years: 14.0, 1.00 (0.54-1.85) oBP × 6-9 years: 12.8, 1.13 (0.58-2.20) oBP × 10-13 years: 15.6, 1.28 (0.69--2.37)
	Clinical vertebral fracture oBP × 2 years: 12.8, 1 oBP × 3-5 years: 15.7, 1.27 (0.71-2.28) oBP × 6-9 years: 17.9, 1.53 (0.84-2.78) oBP × 10-13 years: 16.9, 1.68 (0.95-2.98)
	Any clinical fracture oBP × 2 years: 85.1, 1 oBP × 3-5 years: 89.3, 1.08 (0.87-1.34) oBP × 6-9 years: 93.2, 1.12 (0.89-1.41) oBP × 10-13 years: 110.0, 1.36 (1.10-1.68)*^a^*
**Izano 2020 (** 18)**, USA, retrospective cohort** Exposure: oBP × 5 years vs oBP × 7 years vs oBP × 10 years	Adjusted risk difference per 1000 women (95%CI)
	Hip fracture oBP × 7 years vs oBP × 5years: −2.2 (−20.3 to 15.9) oBP × 10 years vs oBP × 5years: 3.8 (−7.4 to 20.1)
**Wang 2016 (** 46)**, Taiwan, retrospective cohort** Exposure: oBP ≥ 5 years vs oBP < 5 years	Adjusted HR (95%CI)
	Any clinical fracture: 1.49 (0.91-2.45)
	Vertebral fracture: 1.09 (0.63-1.88)
	Nonvertebral fracture: 1.30 (0.45-3.72)

Abbreviations: ALN, alendronate; ETI, etidronate; oBP, oral bisphosphonates; PBO, placebo; RIS, risedronate.

^*a*^Significant but *P*-value unreported.

The evidence on fracture risk varied across differing durations of oBP and fracture type. Extended alendronate for 10 years was associated with a reduced clinical vertebral fracture risk when compared with 5 years of therapy (aRR: 0.45, 95%CI: 0.24-0.85), even when data from patients on subtherapeutic alendronate doses (5 mg) were included (37). In contrast, treatment for 10-13 years was associated with an increased clinical fracture risk compared to 2 years of therapy (adjusted HR: 1.36, 95%CI: 1.10-1.68) (40).

### Adverse events

#### Atypical femoral fractures

Overall, the 4 observational studies suggest oBP use beyond 5 years may be associated with an increased risk of AFF (42), incomplete AFF (iAFF) (39), and atypical fractures (Table 7) (45, 46).

**Table 7:** Harms of extended oral bisphosphonate therapy (>5 years)

Study, country, study design	Outcome
**Canbek 2019 (** 39)**, Turkey, cohort** Exposure: oBP ≤ 4 years vs oBP × 5-9 years vs oBP ≥ 10 years	Age adjusted RR (95% CI)
	iAFF oBP ≤ 4 years vs oBP × 5-9 years: 3.00 (0.64-14.0) oBP ≤ 4 years vs oBP ≥ 10 years: 9.64 (3.29-28.3)*^a^*
**Koh 2017 (** 42)**, South Korea, case-control** Case: AFF (n = 43) Control: no AFF (n = 129)*^b^*	n (%)
	AFF oBP ≥ 5 years: 33 (76.7)
**Park-Wyllie 2011 (** 45)**, Canada, case-control** Case: ST/femoral shaft fracture (n = 716) Control: no ST/femoral shaft fracture (n = 3580)	n (%), aOR (95%CI)
	ST/femoral shaft fracture oBP < 100 days: 42 (5.9), reference oBP × 100 days to 3 years: 349 (48.7), 0.90 (0.48-1.68) oBP × 3-5 years: 204 (28.5), 1.59 (0.80-3.15) oBP ≥ 5 years: 121 (16.9), 2.74 (1.25-6.02)*^a^*
**Wang 2016 (** 46)**, Taiwan, retrospective cohort** Exposure: oBP ≥ 5 years vs oBP < 5 years	Adjusted HR (95%CI)
	ST/diaphyseal fracture 3.10 (0.82-11.73)

Abbreviations: AFF, atypical femoral fracture; iAFF, incomplete AFF; oBP, oral bisphosphonates; ST, subtrochanteric.

^*a*^Significant but *P*-value unreported.

^*b*^Control group included 2 participants on IV zoledronate.

In one study, 76.6% of AFF occurred after more than 5 years of oBP therapy (42). Another study reported elevated iAFF risk among individuals receiving more than 10 years of oBP when compared to 4 years of use (age adjusted RR: 9.64, 95%CI: 3.29-28.3) (39). Compared with individuals exposed to under 100 days of oBP, those on 5 years of therapy demonstrated an increased risk of atypical fracture (aOR: 2.74, 95%CI: 1.25-6.02) (45). However, none of the studies directly compared 5 years of oBP use against extended therapy beyond this threshold.

#### Osteonecrosis of the jaw

No studies specifically examining the impact of extended oBP on ONJ were identified in this review. All retrieved studies reporting ONJ could not be included due to the inclusion of males, individuals with cancer, and intravenous bisphosphonate-use. From the included studies, no cases of ONJ were reported, with 2 studies specifically stating this (37, 41).

## Discussion

This review found contrasting evidence on the benefits and harms of extended oBP beyond 5 years. The ability to draw definitive conclusions is limited by significant methodological and clinical heterogeneity among the included studies that precluded meta-analyses. More than half of the studies were conducted in North America, with reduced representation from Asia (3/11 studies) and none from Africa. Additionally, over half the studies did not report participant ethnicity, reducing generalisability. Variations in oBP type, dose, and duration, as well as differences in statistical methodology, including the use of suboptimal reference groups, further restricted meaningful comparisons across studies.

Nevertheless, this review still offers valuable insights; existing studies provided evidence suggesting that while oBPs might increase BMD (5/5 studies) and reduce clinical vertebral fractures (1/6 studies), there was also an observed increase in atypical fractures (4/4 studies) with extended oBP use.

### Bone mineral density

In clinical practice, alendronate is the most commonly prescribed oBPs (47). Notably, only Black et al's study directly compared BMD outcomes between oBP continuation and discontinuation after 5 years of therapy (20). The global BMD increases were observed despite the pooling of data from patients on subtherapeutic alendronate (5 mg) doses, suggesting that therapeutic doses (10 mg) could yield superior benefits. The dose-dependent effect was reflected by Bone et al's within-group analysis that showed greater BMD increases among participants receiving a 10 mg dose compared to a subtherapeutic 5 mg dose over a 10 year period, especially at the lumbar spine (38). Although Bone et al did not perform intergroup comparisons, the women who discontinued alendronate after 5 years experienced significant reductions in femoral neck, total hip, and distal forearm BMD after 10 years (38).

Evidence from other oBP was less aligned. Among postmenopausal women with at least 2 pre-existing vertebral fractures, extended risedronate use did not yield a significant lumbar spine BMD increase between years 5 and 7 (43). In contrast, etidronate for 7 years produced increased lumbar spine BMD at year 7, albeit when compared to baseline, among women with at least one vertebral fracture (44). Interpretation of these findings is further limited as etidronate is no longer recommended for postmenopausal osteoporosis management due to uncertain efficacy data (48). Additional studies with recommended oBPs, such as alendronate and risedronate (14), are required to provide insight into the role of extended oBPs among women with pre-existing vertebral fractures.

### Osteoporotic fractures

Extended therapy with alendronate for 10 years reduced clinical vertebral fractures compared with 5 years of therapy, even when the analysis pooled data from individuals on subtherapeutic doses (20). However, these findings were inconsistent with the remaining 5 studies reporting vertebral fractures. Notably, Drieling et al and Wang et al reported an increased, albeit nonstatistically significant, vertebral fracture risk associated with oBP therapy beyond 5 years (40, 46). Importantly, the magnitude of these associations did not reach thresholds typically considered clinically meaningful. Potential explanations for these findings include confounding by indication, where individuals at inherently elevated risk of fractures are more likely to be prescribed long-term oBP. Additionally, prolonged exposure could result in over suppression of bone remodeling, potentially increasing fracture susceptibility (49). Interpretation of these findings is further limited by the choice of reference group in Drieling et al, which utilized 2 years of oBP use as the comparator due to its lower fracture incidence (40). There is further reduced clarity on this matter, with the remaining 3 studies finding no significant increase in crude vertebral fracture rates with oBP use beyond 5 years (38, 43, 44).

Among women with pre-existing vertebral fractures, extended oBP use did not significantly reduce subsequent vertebral fracture risk (43, 44). Notably, one of these studies utilized etidronate (44), an oBP now known to have uncertain efficacy (48). The current evidence is insufficient to determine if extended oBP therapy reduces subsequent osteoporotic fractures in women with pre-existing vertebral fractures.

Lastly, Drieling et al reported an increased risk of any clinical fracture with oBPs for 10-13 years when compared to 2 years of therapy, although this association lost statistical significance when oBPs were prescribed for 6-9 years (40). Crucially, this study did not find any significant increase in site-specific fracture risks (40).

In summary, conflicting results and variations in statistical analyses preclude definitive conclusions to be drawn regarding the fracture-reducing benefits of extended oBP therapy.

### Atypical femoral fracture

While the overall trend suggests an increased AFF risk with extended oBP therapy, significant heterogeneity and inconsistent statistical reporting limit the strength of this conclusion. None of the included studies exclusively included individuals with at least oBPs for 5 years, although majority of AFFs occurred beyond this threshold (42, 45, 46).

While one study found an increased age-adjusted risk of iAFF among postmenopausal women on oBPs beyond 10 years compared to those with under 5 years of oBPs, this finding was no longer significant when compared to therapy for 5-9 years (39).

Contradictory results emerged with regard to the association between oBP duration and AFF risk. While one study found an increased odds of AFF with oBPs beyond 5 years when compared to transient use under 100 days (45), another study found no significant difference when utilizing 5 years of oBPs as the cut-off (46). Moreover, these 2 studies included subtrochanteric fractures (ST) in defining atypical fractures, representing more than half (411/716) of the cases in one study (45), and being unreported in another (46). The inclusion of ST fractures could potentially overestimate the atypical fracture cases, because, as proposed by the American Society for Bone and Mineral Research, ST fractures should satisfy certain criteria to be considered atypical (50). Ultimately, this has the potential to impact the reported risk of extended oBPs with uncertain directionality.

Additionally, the relatively small sample size in most studies, each reporting under 200 cases, limits the statistical power, with the exception of Park-Wyllie et al, which included 716 cases (45). Future studies with larger samples focusing specifically on AFF with extended oBPs are essential to determine the magnitude of harm and inform decisions surrounding treatment duration.

### Strength and limitations

To the best of our knowledge, this is the first systematic review to specifically summarize the health outcomes and adverse events associated with extended oBP use beyond 5 years among postmenopausal women. Previous reviews have typically included males (28, 29), employed a shorter treatment duration (19, 28), or pooled data from oral and intravenous bisphosphonates (19, 28, 29). Our review defined extended therapy to be beyond 5 years, in alignment with the initial duration of oBPs recommended by clinical guidelines (11, 51). The deliberate focus on postmenopausal women stems from the known pathophysiological and pharmacokinetic differences conferred by gender and route of bisphosphonate administration (52, 53).

The review was limited by substantial clinical and methodological heterogeneity that precluded the pooling of data to facilitate more robust conclusions to be drawn. Adverse event reporting was limited, with no studies reporting ONJ, a rare but clinically important complication. The lack of ONJ data shifts the review toward focusing on the health outcomes seen in extended oBP therapy. Additionally, results from observational studies are at risk of confounding by indication because women with lower BMD are more likely to receive extended oBP therapy. Consequently, the data of women receiving oBP beyond 5 years may be skewed toward including individuals with more severe osteoporosis, thereby potentially overestimating the fracture risk.

Finally, several potentially relevant studies could not be included in this review as the population comprised both genders, utilized shorter durations to define prolonged oBP use, or included data from patients receiving intravenous bisphosphonates. Despite our best efforts to contact the authors for additional data, insufficient data precluded the inclusion of these reports.

### Implications for practice and policy

High-quality evidence to inform extended oBP-use among postmenopausal women remains limited, highlighting the need for further well-designed studies to address this critical gap. In the interim, decisions regarding oBP therapy beyond 5 years should be guided by structured fracture risk stratification that incorporates clinical risk factors, prior fracture history, BMD, and validated tools such as FRAX®, in conjunction with algorithms developed by international guidelines (12, 15). Until more conclusive evidence is available, physicians should continue to engage patients and provide individualized care through shared decision-making.

## Conclusion

This review found limited and low certainty evidence on the benefits and harms of extended oBP therapy beyond 5 years in the management of postmenopausal osteoporosis. While existing evidence indicates that extended oBP therapy may increase bone density and reduce vertebral fractures, it could also increase atypical fractures. Future high-quality studies with greater statistical power focusing specifically on the impact of extended oBPs are required to validate these findings.

## Data Availability

Data sharing is not applicable to this article as no new datasets were generated or analyzed during the current study.
